# Scalable CNN-based classification of selective sweeps using derived allele frequencies

**DOI:** 10.1093/bioinformatics/btae385

**Published:** 2024-09-04

**Authors:** Sjoerd van den Belt, Hanqing Zhao, Nikolaos Alachiotis

**Affiliations:** Department of Computer Science, Faculty of EEMCS, University of Twente, 7522NB Enschede, The Netherlands; Department of Computer Science, Faculty of EEMCS, University of Twente, 7522NB Enschede, The Netherlands; Department of Computer Science, Faculty of EEMCS, University of Twente, 7522NB Enschede, The Netherlands

## Abstract

**Motivation:**

Selective sweeps can successfully be distinguished from neutral genetic data using summary statistics and likelihood-based methods that analyze single nucleotide polymorphisms (SNPs). However, these methods are sensitive to confounding factors, such as severe population bottlenecks and old migration. By virtue of machine learning, and specifically convolutional neural networks (CNNs), new accurate classification models that are robust to confounding factors have been recently proposed. However, such methods are more computationally expensive than summary-statistic-based ones, yielding them impractical for processing large-scale genomic data. Moreover, SNP data are frequently preprocessed to improve classification accuracy, further exacerbating the long analysis times.

**Results:**

To this end, we propose a 1D CNN-based model, dubbed FAST-NN, that does not require any preprocessing while using only derived allele frequencies instead of summary statistics or raw SNP data, thereby yielding a sample-size-invariant, scalable solution. We evaluated several data fusion approaches to account for the variance of the density of genetic diversity across genomic regions (a selective sweep signature), and performed an extensive neural architecture search based on a state-of-the-art reference network architecture (SweepNet). The resulting model, FAST-NN, outperforms the reference architecture by up to 12% inference accuracy over all challenging evolutionary scenarios with confounding factors that were evaluated. Moreover, FAST-NN is between 30× and 259× faster on a single CPU core, and between 2.0× and 6.2× faster on a GPU, when processing sample sizes between 128 and 1000 samples. Our work paves the way for the practical use of CNNs in large-scale selective sweep detection.

**Availability and implementation:**

https://github.com/SjoerdvandenBelt/FAST-NN

## 1 Introduction

Positive natural selection is a driving factor for the adaption and evolution of a species. The genome of a species that has been affected by positive natural selection contains one or more identifiable regions called selective sweeps. A complete hard selective sweep occurs as a consequence of a beneficial mutation that is spread to the entire population. Modern DNA sequencing methods enable the efficient accumulation of genetic data (Schuster 2008), thereby fueling a rapid increase of sample sizes that results in more precise detection of positive selection (Pavlidis *et al.* 2013). Single nucleotide polymorphisms (SNPs) are typically used for selective sweep detection. An SNP corresponds to a locus along the genome where a mutation has occurred; it can be encoded as a vector of binary states indicating either an ancestral (original) or a derived (mutated) allele at the polymorphic locus across the sample size.

Selective sweeps leave three distinct signatures in the genome that enable their detection: a localized reduction of polymorphisms in a subgenomic region (Smith and Haigh 1974), a shift in the site frequency spectrum (SFS) toward high- and low-frequency derived variants (Braverman *et al.* 1995), and a specific pattern of linkage disequilibrium (LD) where high LD is observed on both sides of the beneficial mutation site while low LD is observed across the selected locus (Kim and Nielsen 2004). Detection methods mainly rely on summary statistics (Alachiotis and Pavlidis 2018) or implement likelihood-based approaches (DeGiorgio *et al.* 2016). However, recent studies have shown that machine learning methods outperform statistical ones in identifying selective sweeps (Flagel *et al.* 2019, Torada *et al.* 2019, Nguembang Fadja *et al.* 2021, Zhao *et al.* 2023a,b) by using convolutional neural networks (CNNs) (LeCun and Bengio 1995) to learn the difference between neutral and selective-sweep regions.

Despite continuous advances in selective sweep detection methods, by virtue of machine and deep learning approaches, the practical and reliable detection of selective sweeps remains challenging. Statistical detection methods can be confounded by genetic factors like demographic changes in population size or migration, as these factors affect patterns of genetic diversity, such as the SFS, similarly to a selective sweep (Weigand and Leese 2018). CNN-based approaches, on the other hand, while generally more robust to confounding factors, do not scale to large data sizes, and require expensive computing resources and hardware accelerators, e.g. GPUs, to run efficiently. CNN-based methods either use summary statistics as features (Kern and Schrider 2018), thereby remaining susceptible to confounding factors but scalable to large sample sizes, or process raw SNP data (Flagel *et al.* 2019, Torada *et al.* 2019, Nguembang Fadja *et al.* 2021, Zhao *et al.* 2023a,b), hence becoming prohibitively compute-intensive; computational complexity increases exponentially with the sample size and the number of SNPs (Visscher *et al.* 2012).

Furthermore, the accurate localization of a selective sweep requires fine-grained genomic scans, which typically rely on sliding-window algorithms with a small step (usually smaller than the window width) to achieve fine detection granularity. While this approach reduces the chances of omitting genomic regions of potential interest from the analysis, it further exacerbates computational costs as it repeats computations during CNN inference for the overlapping regions between consecutive windows. Due to the translation invariance property of CNNs (the ability to recognize patterns in an image regardless of their location), data-reuse optimizations can be applied to avoid redundant computations during CNN inference (Sermanet *et al.* 2013). CNN-based selective sweep classification methods, however, typically reorder SNP data prior to every CNN inference as a preprocessing step (Flagel *et al.* 2019, Torada *et al.* 2019, Zhao and Alachiotis 2023). This improves classification accuracy but precludes data-reuse optimizations because the overlapping regions between consecutive sliding windows differ. Consequently, all corresponding CNN computations need to be performed for every inference. Moreover, data reordering distorts the LD signature of a selective sweep. It might improve classification accuracy when every window/image is processed independently, but, considering that the actual extent of a selective sweep is unknown, distorting the LD pattern independently and possibly in a different way across consecutive windows/images can have a negative effect on detection accuracy.

In this work, we introduce a novel and scalable CNN-based framework for selective sweep classification, dubbed FAST-NN (reversed abbreviation of Neural Network for Tracing Sweeps with Allele Frequencies). FAST-NN overcomes the computational inefficiencies of existing CNN architectures while achieving higher classification accuracy. It is a summary-statistic-free method that learns to identify two selective-sweep signatures, the expected reduction of local polymorphism (Smith and Haigh 1974) and the particular shift of the SFS (Braverman *et al.* 1995), through a vector of derived allele frequencies and a vector of SNP positions (representing SNP density in a region), respectively. Both vectors are 1D and invariant to the sample size, thereby resulting in a scalable solution for large sample sizes. FAST-NN is discovered through an extensive neural architecture search (NAS) that was specifically designed for the proposed compact input data representation. It does not require any data reordering as preprocessing to attain high classification accuracy, allowing the application of data-reuse optimizations for time-efficient, fine-grained, whole-genome scans for selective sweep detection.

In addition, FAST-NN uses early data fusion that improves classification accuracy in the presence of confounding factors (e.g. population bottlenecks, recombination hotspots, and gene flow) by passing SNP positions to the CNN model as input. These positions encode the degree of local polymorphism along the genome, allowing the CNN to infer the degree of reduced polymorphism per subgenomic region. Flagel *et al.* (2019) use late data fusion for selective sweeps, fusing SNP positions with the CNN output through a fully connected layer at the final stage of the classification model. In a fully connected layer, each value is combined with every other, hiding the spatial SNP arrangement and not allowing the CNN to take into account that neighboring positions could be more closely related than distant ones, through higher LD, for instance. Moreover, late fusion prevents the CNN from associating the SNP positions with the raw SNP data. FAST-NN builds upon an early data fusion approach previously used in the classification of recombination hotspots (Chan *et al.* 2018), and adapts it for selective sweep classification. In this implementation, the spatial arrangement of SNP positions is explicitly passed to the CNN model through convolutional layers. By fusing the position data through a convolutional channel, the SNP positions and the raw SNP data are fused at the input of the CNN model, enabling it to account for any patterns that arise from their combination. This approach enables FAST-NN to easily understand the amount of local polymorphism at any particular location within a window, a crucial indicator of selective sweeps.

We evaluated FAST-NN using coalescent simulations under various confounding factors, such as population bottlenecks and recombination hotspots, observing comparable or higher classification accuracy than state-of-the-art CNNs that either use summary statistics or raw SNP data, while being up to two orders of magnitude faster as the sample size increases. Furthermore, to demonstrate the ability of FAST-NN to efficiently learn genomic data patterns from the data it is provided with, we trained FAST-NN using simulated genomic regions characterized by previously validated distributions of fitness effects (DFEs) of deleterious mutations for the Homo Sapiens (Lauterbur *et al.* 2023).

## 2 Materials and methods

### 2.1 Data representation

**Figure 1.**
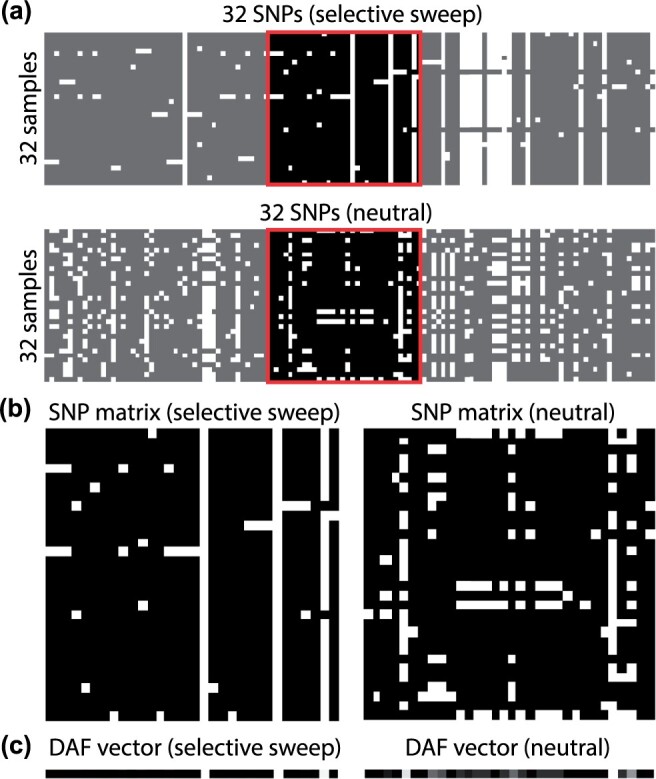
(a) Two 32-SNP windows sampled from larger selective-sweep (top) and neutral (bottom) datasets. (b) and (c) The resulting SNP matrices and their visual representation as derived allele frequency vectors, respectively.

Raw SNP data are represented as a 2D matrix, henceforth referred to as SNP matrix, where rows represent samples and columns represent SNP vectors. Using the Infinite-Sites Model (ISM Kimura [1969]), a value *a_ij_* in the SNP matrix represents the state of the allele at locus *i* of sample *j* and assumes one of two states, “0” or “1,” where “0” indicates the ancestral state and “1” indicates the derived state. The derived allele frequency (DAF) at a locus is determined by the number of derived alleles divided by the total number of samples. The DAF at locus *i* in the SNP matrix can be computed as follows:

(1) $$DAF_{i}=\frac{\sum_{j=0}^{N}{\text{a}}_{ij}}{N},$$

where *N* is the sample size. The DAF vector size is invariant to the sample size. Missing data are handled by reducing the denominator of Equation (1) by the amount of missing alleles per locus, similarly to the way likelihood-based sweep detection methods handle missing data (Pavlidis *et al.* 2013, DeGiorgio *et al.* 2016). Figure 1 illustrates SNP matrices for a neutral segment and a selective sweep (b) extracted from two longer genomic regions (a) and represented as 1D, grayscale DAF vectors (c). A column along the SNP matrix is converted to an allele frequency by counting the number of derived alleles in a column, and dividing over the total number of samples, the result of which can be converted to a pixel value by multiplying by 255.

The amount of polymorphism in a genomic segment can be described by the density of the SNPs, inferred from a vector, *P*, of SNP positions. Flagel *et al.* (2019) use such a position vector as input to the model, which is only possible because the SNP matrix comprises all the SNPs from one simulation. However, when the input to a model is only an SNP window, using the position vector *P* as raw data for training and inference is problematic as the training can be biased by the location of the window in the genome. For example, when a CNN model is trained with data obtained through a number of simulations where the selective sweep is simulated at the same position every time, the model will be biased to learn the position instead of the sweep pattern. To avoid this, relative SNP positions to a single column in the SNP matrix can be used. This approach, however, is not translation invariant and does not allow for any data-reuse optimizations. As an alternative, we use a vector *D* that comprises distances between neighboring SNPs to describe SNP density (Chan *et al.* 2018), which is computed for all positions in an SNP window except the last one as follows:

(2) $$D_{i}=P_{i+1}-P_{i}.$$

*D_i_* for the last SNP is 0. Using *D* creates a translation-invariant solution that is independent of the actual SNP positions in the matrix, avoiding any training bias. Also, the *D* and *DAF* vectors have the same length, allowing their processing as a single matrix.

### 2.2 CNN model architecture

For artificial neural networks (ANNs), there is currently no analytical method to determine the ideal architecture for the problem at hand. While it is a common practice to empirically determine network architectures based on the problem complexity, expected data patterns, and insights from existing architectures, a more rigorous approach deploys an NAS to find the best-performing neural network architecture within a pre-defined search space. Previously, Zhao *et al.* (2023a) performed NAS to discover a suitable network architecture for selective sweep classification (SweepNet), using SNP matrices as input and implementing an iterative process to reduce the number of candidate architectures by optimizing hyperparameters sequentially. Because of the different data representation in our approach, we designed and performed a grid-based NAS to test the entire hyperparameter space. With our 1D, sample-size-invariant SNP representation, the number of operations required to train a single CNN model is significantly reduced, thereby making a full grid-based search computationally feasible.

**Figure 2.**
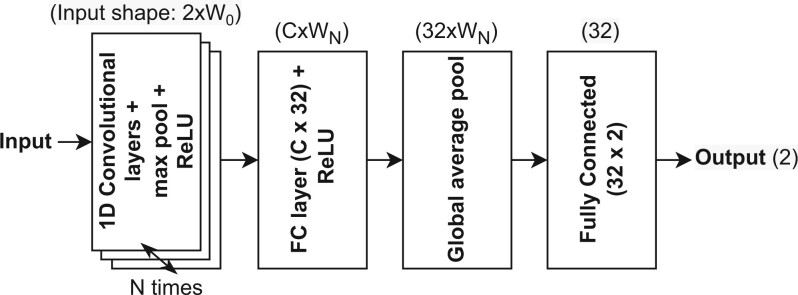
Base model for NAS grid search. The first step is repeated *N* times based on the architecture (input data shape in parentheses).

Figure 2 shows the NAS base model (starting point), and Table 1 describes the NAS hyperparameter space. The base model is derived from SweepNet (Zhao *et al.* 2023a) by omitting the Squeeze-and-Excitation layer (Hu *et al.* 2018) for computational simplicity, and replacing 2D convolutional layers with 1D ones. Each convolutional layer is followed by a max pooling layer with a kernel of width and stride of 2. Due to the pooling layer, the data width reduces after each convolution layer, decreasing the amount of operations and increasing the receptive field of the model.

**Table 1.** List of hyperparameters and parameter space (empirically determined) for grid-based NAS, resulting in 180 candidate models in total.

Hyperparameter	Options	Models
Convolutional layers	2, 3, 4, 5	4
Convolution channels	8, 16, 32, 64, 80	20
Kernel width	2, 3, 4	60
Kernel stride	1, 2, 3	180

### 2.3 Memory-efficient data format

CNN-based methods that use raw SNP data for selective sweep detection encode the SNP matrix as an image (Flagel *et al.* 2019, Torada *et al.* 2019, Nguembang Fadja *et al.* 2021, Zhao *et al.* 2023a), with each pixel representing an allele. This approach is inefficient in terms of memory usage; it allocates a byte per pixel per channel under the Red-Green-Blue (RGB) or grayscale color models, whereas the encoded information is only one bit (Kimura 1969), or two bits if missing data and/or N characters are used. Moreover, distances between neighboring SNPs that exceed 255 base pairs and derived allele frequencies for sample sizes exceeding 255 samples cannot be precisely encoded in 8-bit channels (RGB).

To efficiently and accurately store SNP data, allele frequencies, and SNP distances, FAST-NN uses a binary-data representation that encodes 8 allele states per byte and stores allele frequencies and SNP distances as single-precision floating-point numbers. When raw data and SNP distances are used, the total number of bytes, *B*, is calculated by Equation (3):

(3) $$B_{raw}=W\times \left(4+\left\lceil \frac{N}{8}\right\rceil \right),$$

where *W* is the matrix width (window width) and *N* is the sample size. When allele frequencies and SNP distances are used, the total number of bytes is calculated by Equation (4):

(4) $$B_{daf}=W\times (4+4).$$

Notice that *B_daf_* is not a function of the sample size *N*. Equations (3) and (4) show that the binary format is more memory efficient for samples sizes larger than 32, thus reducing data loading time. For the 1000 Genomes project (Auton *et al.* 2015) total sample size (2504 samples), for instance, the binary representation of allele frequencies reduces the data size by 97.5%.

## 3 Evaluation

### 3.1 Experimental setup

To evaluate FAST-NN, we generated neutral coalescent simulations and simulations with a selective sweep at the center of the simulated region under three demographic models that confound selective sweep detection (population bottlenecks, migration, and recombination hotspots) using the software tools ms (Hudson 2002), mssel (kindly provided by R. R. Hudson), mbs (Teshima and Innan 2009), and msHOT (Hellenthal and Stephens 2007). We assumed that the present-day population size is 50 000 diploid genomes. Table 2 provides the simulation parameters per dataset. Each dataset consists of a training set, a validation set, and a test set, comprising 1700, 300, and 2000 simulations, respectively, with each of the three sets evenly split into neutral simulations and simulations with a selective sweep. The sample size is 128, and a 128-SNP window is extracted from the center of each simulation, resulting in a 128 × 128 SNP matrix per simulation.

**Table 2.** Evaluation datasets and their simulation parameters.

Confounding factor	Software	Parameters	Values
Mild bottleneck (D1) Severe bottleneck (D2)	Neutral: ms Selective: mssel	Severity (-eN) Duration (-eN) Beginning (-eN) Selection coefficient (-s) Sweep start time (-t)	0.5 (D1), 0.005 (D2) 0.001 (D1), 0.002 (D2) 0.1 (D1), 0.01 (D2) 0.02 0.016
Recent migration (D3) Old migration (D4)	Neutral: ms Selective: mssel	Population join time (-ej) Selection coefficient (-s) Sweep start time (-t)	0.003 (D3), 3 (D4) 0.02 0.005
Recombination hotspot Low intensity (D5) High intensity (D6)	Neutral: msHOT Selective: mbs	Hotspot intensity (-v) Hotspot region size (-v) Selection coefficient (-s) Sweep start time (-t) Mutation rate (-t) Recombination rate (-r)	2 (D5), 20 (D6) 5 kb 0.02 0.005 2000 2000

FAST-NN (https://github.com/SjoerdvandenBelt/FAST-NN) is realized in Pytorch (Paszke *et al.* 2019). We initially compare performance with SweepNet (Zhao *et al.* 2023a) that inspired the base model used in NAS (Section 2.2), in terms of classification accuracy and training/inference time. Thereafter, we benchmark FAST-NN against other CNNs for selective sweeps, including neural models that use summary statistics. FAST-NN and SweepNet were trained in mini-batches of size 8 for 100 epochs at a learning rate of $0.5\times 10^{-3}$, which empirically resulted in reliable training. The Adam optimizer (Kingma and Ba 2014) was used, and the model with the highest validation accuracy across all epochs was reported. When training using the SNP matrix as input, the rows of the matrix are randomly shuffled, increasing the diversity of the training dataset to reduce the effects of overfitting. Execution times were measured on a single CPU core (Intel Xeon at 2.1 GHz running Ubuntu 20.04) and on a GPU (Nvidia A40).

### 3.2 Fusing raw data with positions

To evaluate the effect of fusing SNP positions with the raw data, we created two variants of the previously established SweepNet model that process SNP distances between neighboring SNPs. This evaluation was based on SweepNet before deriving the base model for NAS (Section 2.2) to reduce the search space. The first variant, henceforth referred to as SN-FC-late, introduces SNP distances through a fully connected layer, the output of which is appended to the input of the final fully connected layer of SweepNet (late fusion), as proposed by Flagel *et al.* (2019). The second variant, henceforth referred to SN-Conv-early, employs an additional input channel added to the very first convolutional layer (early fusion).

**Figure 3.**
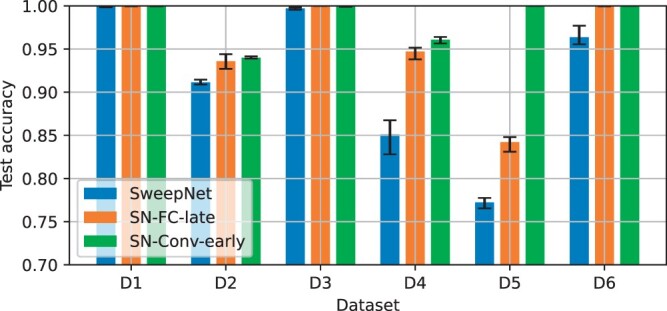
Effect of fusing SNP positions with raw data on SweepNet inference. Average accuracy is shown, with error bars representing the range across 10 training runs. Early fusion (SN-Conv-Early) achieves higher test accuracy than late fusion (SN-FC-late).

Figure 3 compares the test accuracy of the two data-fusion variants with the initial network. Every network was trained 10 times for each dataset. The bar chart indicates the mean accuracy of the 10 models per dataset, and the error bars indicate the least and the best performing models. It can be observed that data fusion improves test accuracy across all tested confounding factors, with SweepNet-CONV-Early outperforming SweepNet-FC-Late. Especially in the case of a recombination hotspot with low intensity (D5), SweepNet had an average test accuracy of 0.77 while SweepNet-FC-Late and SweepNet-CONV-Early achieved 0.84 and 1.0, respectively. Integrating SNP positions via convolutional layers (early) instead of a separate fully connected layer (late), improves model performance by exposing the spatial arrangement of positions, making it easier for the model to learn local spatial patterns, e.g. the SNP density in genomic region.

### 3.3 Neural architecture search

NAS for FAST-NN was based on the dataset with old migration (D4) as one of the more challenging cases. As shown in Fig. 3, severe population bottlenecks (D2) confound classification as well. The NAS base model was derived from SweepNet, which was previously discovered through a dedicated NAS that incorporated simulated datasets with severe population bottlenecks (Zhao *et al.* 2023b). By further optimizing the CNN model for old migration, NAS for FAST-NN resulted in a new model with better performance under the most challenging confounding factors.

**Figure 4.**
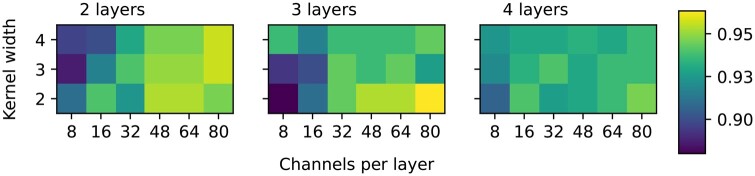
Validation accuracy of candidate 1D convolutional models for dataset D4 (old migration).

**Figure 5.**
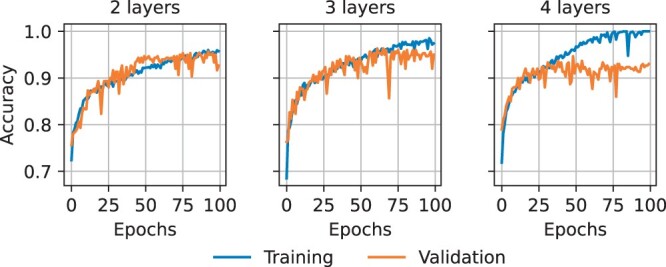
Training and validation accuracy of the best model per number of convolution layers. The models with more layers overfit.

Figure 4 shows the highest validation accuracy for each candidate model. The model with 3 convolutional layers, with each layer consisting of 80 channels and a kernel width of 2, achieved the highest validation accuracy (0.963). Models with less channels per convolutional layer reached a lower validation accuracy, as, most likely, they do not have enough parameters to capture the complex patterns of a selective in the presence of confounding factors. Models with 4 convolutional layers do not outperform the 3-layer models either, as they are more prone to overfitting. Figure 5 illustrates the training and validation accuracy of the best model for each number of convolutional layers. As can be observed, the 4-layer model overfits, plateauing at a lower validation accuracy, while the 3-layer model slightly overfits as well, yet achieving a higher validation accuracy than the best 2-layer model. Since the model state is saved after the epoch with the highest validation accuracy, we use the 3-layer model state before it overfits.

### 3.4 Classification using DAFs

**Figure 6.**
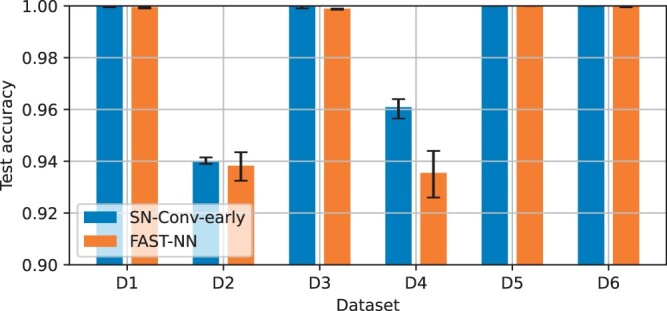
Comparison of average mean accuracy and accuracy range (across 10 training runs) between FAST-NN and SN-Conv-early.

We compared FAST-NN with SweepNet with early data fusion (SN-Conv-early) to quantify the impact of using derived allele frequencies (DAFs) on classification accuracy (Fig. 6). For datasets D1, D3, D5, and D6, both models achieve test accuracies between 0.998 and 1.0. For the challenging confounding factors of a severe bottleneck (D2) and old migration (D4), both models have comparable performance: SN-Conv-Early achieves maximum accuracies of 0.942 and 0.964, respectively, while FAST-NN achieves 0.944 on both. FAST-NN learns the SFS sweep signature from the DAF vector and does not need to process the raw data to reach comparable classification accuracy with SweepNet.

Table 3 provides execution times for training and testing when FAST-NN and SN-Conv-Early process raw data. On the CPU, FAST-NN trains between 17.73× and 259.0× faster while performs inference between 20.52× and 242.7× faster, when the sample size ranges from 64 to 1000 samples. On the GPU, the speedups range between 1.23× and 6.15× for training, and between 1.0× and 3.85× for testing, both increasing with the sample size. Because both models process raw data under the same format (images or binary), this speedup comparison quantifies the performance improvements attributed to the different network architecture. The reduced GPU speedup can be attributed to the data transfers to/from the GPU. Due to the short execution time of FAST-NN, the communication overhead becomes a bottleneck. This unfavorable computation-to-communication ratio generally occurs when performing small computational tasks using hardware accelerators with a dedicated memory space. Moving data in larger batches reduces the number of transfers and can increase the GPU speedup.

**Table 3.** Training time per epoch and inference time for SN-Conv-early and FAST-NN for SNP matrices of width 128 and height 64, 128, and 1000 using various data loading methods.

Sample size	Data type, format	CPU/GPU	SN-Conv-early		FAST-NN		Speedup (x)	
			Train	Test	Train	Test	Train	Test
64	Raw, images	CPU	51.25	33.85	2.89	1.65	17.73	20.52
		GPU	2.09	1.61	1.70	1.61	1.23	1.0
	Raw, binary	CPU	45.33	25.91	2.31	1.14	19.62	22.73
		GPU	2.19	1.11	1.31	1.08	1.94	1.03
128	Raw, images	CPU	106.27	63.81	3.56	2.00	29.85	31.91
		GPU	3.26	2.56	2.14	2.53	1.52	1.01
	Raw, binary	CPU	95.50	54.10	3.19	1.35	29.94	40.01
		GPU	4.27	1.88	2.16	1.64	1.98	1.15
1000	Raw, images	CPU	1387	779	9.95	7.05	139.4	110.5
		GPU	30.57	13.13	8.31	7.73	3.68	1.70
	Raw, binary	CPU	1300	711.2	5.02	2.93	259.0	242.7
		GPU	25.57	13.05	4.16	3.39	6.15	3.85

**Figure 7.**
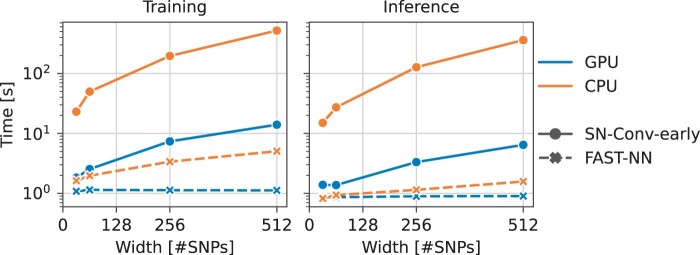
Training and inference times for increasing window width. Time (*y*-axis) is displayed on a logarithmic scale.

Table 4 compares the fastest FAST-NN (DAF, binary) and SN-Conv-early (raw data, binary) implementations. Using DAFs leads to higher speedups than processing raw data as the sample size increases. Notice the slowdown for GPU testing with 64 samples, due to an unfavorable computation-to-communication ratio caused by the transfer overhead and the limited amount of computation on the GPU. Figure 7 shows execution times for increasing window widths, up to 512 SNPs. It is observed that FAST-NN scales better than SN-Conv-early for wider genomic segments.

**Table 4.** Performance comparison (speedups) between the fastest FAST-NN (DAF, binary) and SweepNet (raw data, binary) implementations.

CPU/GPU	64 samples		128 samples		1000 samples	
	Train	Test	Train	Test	Train	Test
CPU	19.29	22.15	38.2	53.6	586.2	697.1
GPU	1.26	0.85	2.35	1.42	16.4	11.2

### 3.5 Comparison with other methods

We benchmarked FAST-NN against the CNN-based selective sweep classification methods ImaGene (Torada *et al.* 2019), diploS/HIC (Kern and Schrider 2018), SweepNet (Zhao *et al.* 2023a), and the model of Nguembang Fadja *et al.* (2021). All methods under comparison process raw data as input to a CNN, except for diploS/HIC that uses 12 summary statistics and FAST-NN that uses derived allele frequencies. To avoid overfitting, we trained each model for 10 epochs, using extended training datasets (simulation parameters provided in Table 2) comprising 50 000 neutral simulations and 50 000 simulations with a selective sweep. We have also compared FAST-NN with non-machine-learning summary statistics that are commonly used as neutrality tests in terms of accuracy and execution time (Supplementary Section S1).

**Figure 8.**
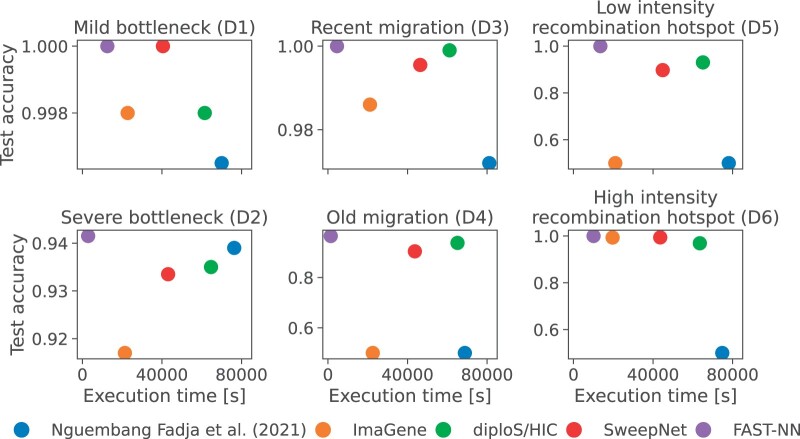
Total time (preprocessing, training, and inference) and test accuracy for various CNN-based sweep classification methods.

Figure 8 shows the total CPU time for preprocessing, training, and inference per model, and the corresponding test accuracy. It can be observed that FAST-NN outperforms all other methods in terms of execution time. Moreover, for all evolutionary scenarios, the accuracy of FAST-NN is either on par with or better than the other methods. The accuracy of the summary-statistic-based diploS/HIC is at least 0.935 for the datasets simulating population bottlenecks (D1 and D2), at least 0.938 for the datasets simulating migration (D3 and D4), and at least 0.931 for the datasets simulating recombination hotspots, while FAST-NN achieves accuracies of 0.942, 0.965, and 1.0, respectively. FAST-NN, however, is between 4.9 and 45.9 times faster than diploS/HIC, depending on the dataset. Across all datasets, ImaGene achieved the second shortest execution time, but it did not train with the datasets simulating old migration (D4) and a low-intensity recombination hotspot (D5). For the datasets that ImaGene trained on successfully, it achieved accuracy between 0.917 (severe bottleneck, D2) and 0.998 (mild bottleneck, D1), while FAST-NN achieved accuracy in the range of 0.942 (D2) to 1.0 (D1).

### 3.6 Human chromosome 1

To showcase the ability of FAST-NN in learning genomic data patterns, we generated FAST-NN models using training datasets simulating validated DFEs of deleterious mutations for the Homo Sapiens under different demographic models. We used stdpopsim (Lauterbur *et al.* 2023), a library of standard population genetic simulation models, and employed SLiM (Haller and Messer 2019) as its simulation engine. We generated two datasets, each comprising 1000 simulations of human chromosome 1 (sample size 100) for training and 100 simulations for testing. For the first dataset, we used the OutOfAfrica-3G09 demographic model (Gutenkunst *et al.* 2009), the PyrhoCEU_GRCh38 genetic map (Spence and Song 2019), inferred using individuals from the CEU (Northern Europeans from Utah) population from Phase 3 of the 1000 Genomes Project (Auton *et al.* 2015), and the Gamma_K17 DFE of deleterious mutations (Kim *et al.* 2017). For the second dataset, we used the Africa-1T12 demographic model (Tennessen *et al.* 2012), the PyrhoYRI_GRCh38 genetic map (Spence and Song 2019), inferred using individuals from the YRI (Yoruba in Ibadan, Nigeria) population from Phase 3 of the 1000 Genomes Project (Auton *et al.* 2015), and the Gamma_H17 DFE of deleterious mutations (Huber *et al.* 2017). The OutOfAfrica-3G09 model of Gutenkunst *et al.* [2009] is a three-population model that specifies the out-of-Africa bottleneck and the subsequent divergence of the European and Asian populations, while the Africa-1T12 model is a simplified version of the Tennessen *et al.* (2012) model with only the African population specified (expansion from the ancestral population and recent growth). Table 5 provides a comparison of execution times for training and testing, and test accuracy, processing 128-SNP windows. FAST-NN achieves comparable accuracy with SweepNet with early fusion (SN-FC-Early) while training 41× faster, on average, and performing inference 2.8× faster, on average.

**Table 5.** Comparison of CPU execution time (s) and test accuracy on simulations of human chromosome 1 (sample size 100, 128-SNP windows).

Genetic map	PyrhoCEU_GRCh38			PyrhoYRI_GRCh38		
Demographic model	OutOfAfrica-3G09			Africa-1T12		
Dist. of fitness effects	Gamma K17			Gamma H17		
	Train	Test	Acc	Train	Test	Acc.
SweepNet	3578	17.5	0.75	3840	16.2	0.74
SN-FC-Early	4144	17.9	0.97	4551	17.0	0.99
FAST-NN	108	6.1	0.97	105	6.5	0.99

## 4 Discussion and conclusion

In this work, we demonstrated the effectiveness of using derived allele frequencies (DAF) and SNP density as input to a CNN called FAST-NN for classifying genomic regions with a hard, complete selective sweep and genomic regions characterized by DFEs for deleterious mutations. Evaluating various evolutionary scenarios of confounding factors for selective sweeps, we showed that FAST-NN exhibits equal or better classification accuracy than state-of-the-art CNN-based methods. The performance gain is attributed to a careful model selection using NAS, combined with effective fusion of DAF data and SNP density data, from which FAST-NN quantifies two known selective-sweep signatures, the expected shift in the SFS toward low- and high-frequency derived variants, and the local reduction of diversity, respectively.

Because the size of the DAF and SNP-distance vectors (1D arrays) is constant with respect to the number of samples, FAST-NN considerably reduces processing time, representing a practical tool to analyze large-scale genomic datasets. The 1D data representation has a significant impact on computational efficiency; the complexity of 2D convolution on an *N *×* N* matrix with a *K *×* K* kernel is $˜O(N^{2}K^{2})$, whereas the complexity of 1D convolution on a 1D vector of length *N* with kernel length *K* is only ˜O(NK) (Kiranyaz *et al.* 2021). Furthermore, only using derived allele frequencies and pairwise SNP distances as input to the CNN reduces the amount of data that need to be stored and passed to the model (in comparison with raw data or various summary statistics), yielding an efficient solution for increasing dataset sizes.

FAST-NN does not require data reordering as a preprocessing step, thereby further increasing efficiency and its applicability for large-scale genomic data. It enables seamless integration into efficient sliding-window approaches, such as the method described by Sermanet *et al.* (2013) for exploiting data reuse in overlapping windows, which requires that the spatial arrangement of the input data to the CNN between consecutive windows is not distorted. Our work facilitates the practical application of CNNs in large-scale selective sweep detection.

## Supplementary Material

btae385_Supplementary_Data

## Data Availability

The data underlying this article are available in figshare, at https://dx.doi.org/10.6084/m9.figshare.26139454.
