# Supplementary material for: Scalable CNN-based classification of selective sweeps using derived allele frequencies
Source: Bioinformatics. 2024 Sep 4;40(Suppl 2):ii29–36. doi: 10.1093/bioinformatics/btae385 (PMC11373383; doi:10.1093/bioinformatics/btae385)
Supplement: btae385_Supplementary_Data [file btae385_supplementary_data.zip › ECCB2024_FAST-NN_Supplementary_File.pdf]

Bioinformatics, 2024, 1–1
doi: DOI HERE
Advance Access Publication Date: Day Month Year
Paper

Scalable CNN-based classification of selective sweeps using derived allele frequencies (Supplementary File)

Sjoerd van den Belt, Hanqing Zhao and Nikolaos Alachiotis*

Faculty of EEMCS, University of Twente, Drienerlolaan 5, 7522NB, Enschede, The Netherlands

*Corresponding author. n.alachiotis@utwente.nl

FOR PUBLISHER ONLY Received on Date Month Year; revised on Date Month Year; accepted on Date Month Year

Supplementary Section 1: Comparison with summary statistics

We compared the performance of FAST-NN with the classification performance of summary statistics used for sweep detection. Tajima's D (Tajima, 1989), Fu and Li's D and F (Fu and Li, 1999) and Rozas' R_2 (Ramos-Onsins and Rozas, 2002) are computed using PopGenome (Pfeifer et al., 2014). PopGenome is a versatile tool for statistical analysis of population genomics, written in R. The μ statistic (Alachiotis and Pavlidis, 2018) is a summary statistic that combines each of the three signatures of selective sweeps: localized reduction of polymorphisms, a shift in the site frequency spectrum, and a specific pattern in linkage disequilibrium. The μ statistic is efficiently computed by the open-source software RAiSD, which is implemented in C.

Table 1 shows the maximum classification accuracy attained using each of the summary statistics, and using FAST-NN trained on 1,700 simulations. The μ statistic outperforms the other statistics for each of the datasets, except for the severe population bottleneck, where Fu and Li's D and the μ statistic achieve an accuracy of 0.9215 and 0.92, respectively. FAST-NN has an accuracy that is greater or equal to each of the summary statistics for all evaluated datasets except D1, where FAST-NN has a single misclassification and the μ statistic has a perfect score. Table 2 shows the execution time of PopGenome and RAiSD, along with the inference time of FAST-NN. All runs are measured on a single CPU core (Intel Xeon at 2.1 GHz running Ubuntu 20.04). When evaluating PopGenome, only the processing time of the function that computes the summary statistic is included in the execution time. Running PopGenome computes all supported summary statistics for testing neutrality in one run. PopGenome can run in fast mode, which speeds up the computation significantly, but it does not support computing Rozas' R_2. RAiSD outperforms PopGenome in terms of execution time, due to its efficient C implementation. The execution time of RAiSD is greater than the inference time of FAST-NN on a CPU, taking an average of 1.87 seconds and 1.24 seconds, respectively. However, using FAST-NN requires training the model, which takes an average of 3.19 seconds per epoch when training on 1,700 simulations.

References

N. Alachiotis and P. Pavlidis. RAiSD detects positive selection based on multiple signatures of a selective sweep and SNP vectors. *Communications biology*, 1(1):79, 2018.

Y.-X. Fu and W.-H. Li. Coalescing into the 21st century: An overview and prospects of coalescent theory. *Theoretical Population Biology*, 56(1):1–10, 1999.

B. Pfeifer, U. Wittelsburger, S. E. Ramos-Onsins, and M. J. Lercher. PopGenome: an efficient Swiss army knife for population genomic analyses in R. *Mol Biol Evol*, 31(7): 1929–1936, 2014.

S. E. Ramos-Onsins and J. Rozas. Statistical Properties of New Neutrality Tests Against Population Growth. *Molecular Biology and Evolution*, 19(12):2092–2100, 2002.

F. Tajima. Statistical method for testing the neutral mutation hypothesis by DNA polymorphism. *Genetics*, 123(3):585–595, 1989.

© The Author 2024. Published by Oxford University Press. All rights reserved. For permissions, please e-mail: journals.permissions@oup.com

Table 1. Classification accuracy of neutrality tests using various summary statistics, and using FAST-NN. Each dataset consists of 1,000 neutral simulations and 1,000 simulations featuring a selective sweep. Each simulation is classified by a window of 128 SNPs, where the window is centered around the selective sweep, when one is present.

Dataset	Tajima's D	Fu and Li's F	Fu and Li's D	Rozas' $\mathbf{R_2}$	μ statistic	FAST-NN
Mild population bottleneck (D1)	0.987	0.9845	0.954	0.987	1.0	0.9995
Severe population bottleneck(D2)	0.8565	0.9165	0.9215	0.861	0.92	0.9435
Recent migration (D3)	0.894	0.848	0.663	0.894	0.978	0.999
Old migration (D4)	0.706	0.7435	0.706	0.7135	0.854	0.944
Low intensity recombination hotspot (D5)	0.609	0.613	0.599	0.608	0.708	1.0
High intensity recombination hotspot (D6)	0.513	0.508	0.513	0.5135	0.9035	1.0

Table 2. Execution time (seconds) of the tools used to compute the summary statistics in table 1, and execution time to perform inference using FAST-NN. Running PopGenome in fast mode computes Tajima's D, Fu and Li's F, and Fu and Li's D. Running PopGenome in normal mode also computes these statistics, with the addition of Rozas' $\mathbf{R_2}$. RAiSD computes the μ statistic.

Dataset	PopGenome	PopGenome (fast)	RAiSD	FAST-NN
Mild population bottleneck (D1)	10.44	4.00	1.87	1.22
Severe population bottleneck (D2)	10.68	4.17	2.17	1.22
Recent migration (D3)	10.23	4.01	1.79	1.19
Old migration (D4)	10.61	3.98	1.79	1.34
Low intensity recombination hotspot (D5)	10.49	4.13	1.75	1.23
High intensity recombination hotspot (D6)	10.76	4.11	1.82	1.25
